# Pilot project: easy read psychiatry clinic appointment outcome letters

**DOI:** 10.1192/bjo.2021.586

**Published:** 2021-06-18

**Authors:** Anu Sharma, Indermeet Sawhney

**Affiliations:** 1 Saffron Ground, Ditchmore lane; 2 Tekhnicon House, Springwood Drive

## Abstract

**Aims:**

To improve communication with patients and carers by sharing information in an easily comprehensible manner.

**Background:**

According to the department of health guidelines, there is legal requirement to provide copies all clinical correspondence to the patients. Therefore, after any clinic review, letters summarizing the consultation are sent out to GP and patients are copied in. However, these are not very meaningful for patients with special needs, as they struggle to comprehend information. Previous studies have shown that patients with learning disability would prefer letters in a simple language and would also like to participate in the decision making process. According to Accessible Information Standard, we have a legal obligation to deliver information to our service users in an easily understandable manner. We undertook a quality improvement pilot project of easy read templates to improve the understanding of patients and their carers/families.

**Method:**

A standard easy read template was co-produced after collecting feedback from different service users and clinicians. Pictures were incorporated into the questionnaire to facilitate understanding. We collected reviews over a period of 2 months from Nov 2019- Dec 2019. This proforma did not replace the routine clinic letter send out to the GP and the patients. This easy read template began with the introduction of the doctor (with photograph) and it encompassed mental health, physical health, current medication (and the benefits and side effects if any) and changes of medication. It also included epilepsy and the risks (risks to self and to others), vulnerability, behaviours of concern and the day-to-day activities that a service user engages in and finally about the plan formulated at the end of the consultation. At the same time, there was a separate form (with self-explanatory pictures), which collected feedback about the above mentioned appointment outcome review form.

**Result:**

Templates were handed out to 65 patients and carers, and 60 completed the form. All patients found the template useful and helpful, mainly because it was easily comprehensible, with pictures, and also “provided instant updates”.

**Conclusion:**

This easy read template improves patients’ understanding and participation in the clinic review. This contributes to greater patient satisfaction. As Specialist Learning Disability services, we need to ensure that information is imparted to the patients and the carers in an easily understandable manner and this easy read template should be incorporated in the routine clinic practice.

